# Comparative study on the impact of ‘Infographic versus video feedback’ on enhancing students’ clinical skills in basic life support

**DOI:** 10.1186/s12909-024-05763-x

**Published:** 2024-07-19

**Authors:** Kheizaran Miri, Ali Yaghoubi, Sadaf Kholousi, Mahdi Yousofzadeh, Alireza Zanganeh, Mehdi Gharayi, Mohammad Namazinia

**Affiliations:** 1 Department of Nursing, School of Nursing and Midwifery, Torbat Heydariyeh University of Medical Sciences, Torbat Heydariyeh, Iran; 2 Student Research Committee, Torbat Heydariyeh University of Medical Sciences, Torbat Heydariyeh, Iran

**Keywords:** Cardiopulmonary resuscitation, Basic Life Support, Infographics, Video feedback

## Abstract

**Background:**

Effective cardiopulmonary resuscitation (CPR) training for nursing students is crucial for improving patient outcomes in cardiac arrest scenarios. This study assesses the impact of infographic versus video feedback on enhancing nursing students’ clinical skills in Basic Life Support (BLS).

**Methods:**

In a randomized controlled setting, 76 nursing students at Torbat Heydariyeh University of Medical Sciences were divided into two groups: one received infographic-based education and the other video feedback training. Pre- and post-intervention assessments measured knowledge and skill retention using validated questionnaires.

**Results:**

Post-training, the infographic group showed significantly higher knowledge scores, while the video feedback group exhibited greater improvement in CPR skill performance. No significant differences were noted in pre-training assessment scores between the groups.

**Conclusion:**

Infographic-based education enhances BLS knowledge retention, and video feedback improves practical CPR skills. This suggests potential benefits of a combined infographic and video feedback approach for optimizing CPR training outcomes, addressing a critical need in medical education.

## Introduction

Cardiopulmonary Resuscitation (CPR) is a critical skill for healthcare providers, particularly in in-hospital settings where cardiac arrests are common and often witnessed by medical staff. Despite the prevalence of in-hospital cardiac arrests and the importance of high-quality CPR, studies have shown that even when performed by trained professionals in hospitals, CPR quality is often suboptimal [1–3].

In-hospital cardiac arrest (IHCA) remains a significant challenge in healthcare, with survival rates varying widely between institutions. Recent data indicate that survival to hospital discharge following IHCA ranges from 10 to 25%, highlighting the urgent need for improved CPR training and performance among healthcare providers [4, 5].

The quality of CPR delivered by healthcare providers, particularly nurses who are often first responders in hospital settings, is crucial in determining patient outcomes. Research has demonstrated that adherence to CPR guidelines, including proper compression depth, rate, and chest recoil, significantly impacts survival rates [6–8]. However, despite widespread training and certification, healthcare providers often struggle to consistently meet these standards during actual resuscitation efforts [7–10].

Traditional CPR training methods, which typically involve theoretical classes and practical demonstrations on mannequins, have shown limitations in ensuring long-term skill retention and quality performance. These methods often fail to provide immediate, objective feedback on CPR quality, which is crucial for skill improvement [11, 12].

Recent studies have explored the use of real-time CPR feedback devices that provide immediate data on compression rate, depth, and recoil. These devices have shown promise in improving CPR quality during actual resuscitations [13–16]. However, such devices are not always available in training settings or may be cost-prohibitive for widespread use in educational programs.

In light of these challenges, there is a growing interest in exploring alternative, cost-effective methods to enhance CPR training for healthcare providers, particularly nursing students. Two such methods that have gained attention are the use of infographics for knowledge retention and video feedback for skill improvement.

Infographics, which combine visual elements with text to present complex information in an easily digestible format, have shown potential in enhancing learning and retention of medical knowledge [17, 18]. Video feedback, on the other hand, allows learners to observe and critique their own performance, potentially leading to improved technique [19, 20].

While these methods differ from real-time CPR quality feedback devices, they offer potential advantages in terms of accessibility and cost-effectiveness in educational settings. However, their comparative effectiveness in improving CPR knowledge and skills among nursing students has not been thoroughly investigated.

Therefore, this study aims to compare the effect of infographic-based education versus video feedback on the CPR knowledge and skills of nursing students. By exploring these alternative training methods, we hope to identify effective, accessible strategies to enhance CPR education and ultimately improve the quality of in-hospital resuscitation efforts.

## Method

### Study design

**Fig. 1:**
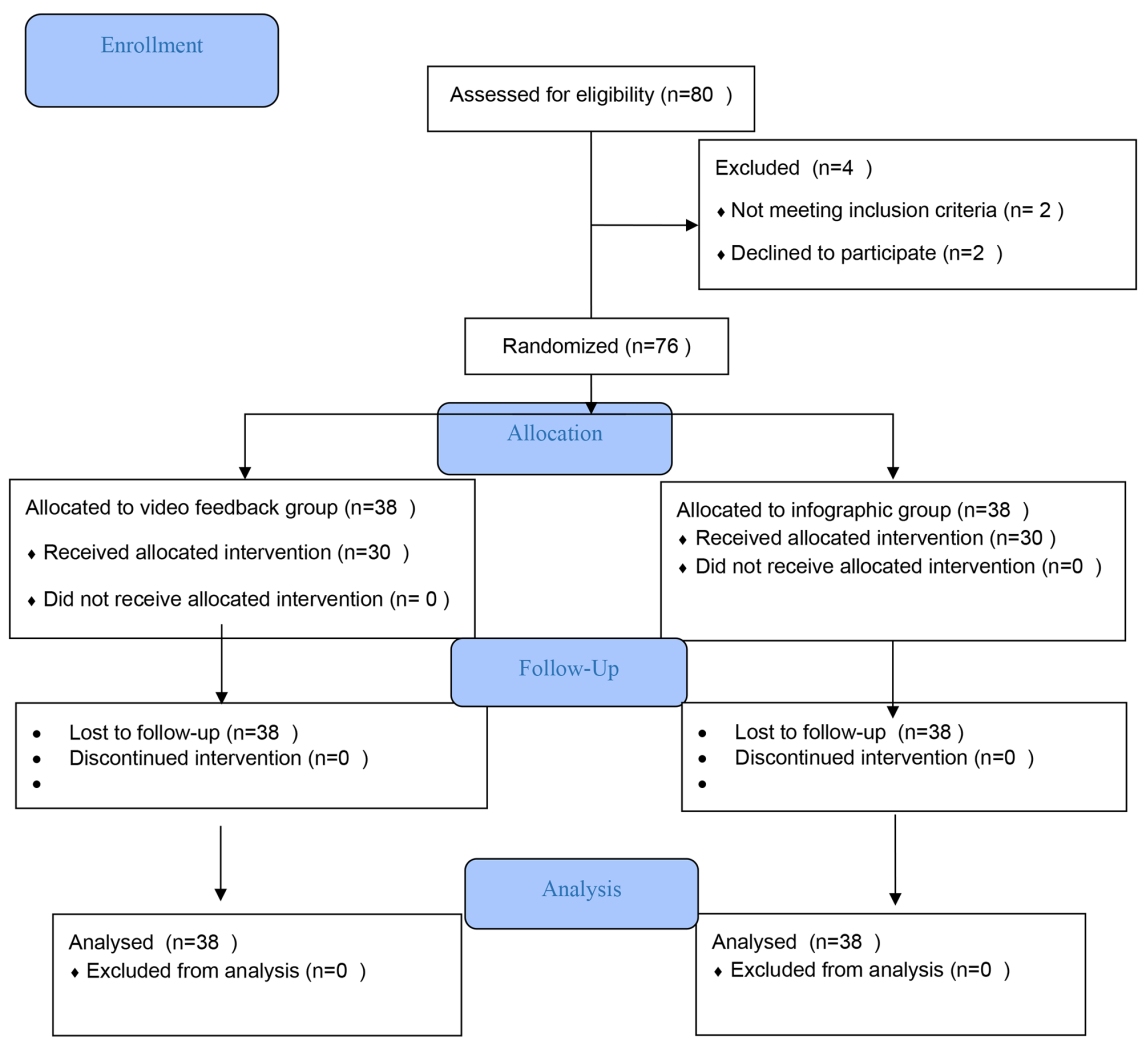
Flow diagram

This study was carried out on nursing students of Torbat Heydariyeh University of Medical Sciences, Iran, in the year 2023, employing a pre-test and post-test design (Fig. 1).

### Participants

The study group consisted of 76 undergraduate nursing students from the Nursing and Midwifery School of Torbat Heydariyeh. Inclusion criteria included nursing students who were studying in the fifth semester or above and had consented to participate in the study. Exclusion criteria were nursing students who were unwilling to participate, absence in any of the intervention sessions, and those who, for reasons such as acute stress, did not have the necessary mental or psychological readiness to perform cardiopulmonary resuscitation skills.

### Outcomes

Data were collected using a demographic information questionnaire, a questionnaire assessing knowledge and awareness about Adult BLS, and a questionnaire assessing cardiopulmonary resuscitation skills in adults.

The demographic information questionnaire consisted of six questions regarding age, average university grades, interest in nursing, work experience in hospitals, and history of performing cardiopulmonary resuscitation. This questionnaire was administered through interviews with the participants. The researcher created the questionnaire, and its validity was verified through content validity using the corrective opinions of ten faculty members from the Nursing-Midwifery School of Torbat Heydariyeh University of Medical Sciences.

Adult BLS Knowledge and Awareness Questionnaire: This researcher-created questionnaire was based on the American Heart Association’s 2022 guidelines. It contains 20 questions about the basic principles of life support (BLS). These questions cover topics such as recognizing signs of cardiac-respiratory arrest, assessing the victim’s level of responsiveness and vital signs, placing the victim in the appropriate position, asking for help, determining the number and depth of chest compressions during cardiac massage, managing the airway initially, performing artificial respiration, and the use of AED. Scores on this questionnaire range from zero to 20, with higher scores indicating nursing students’ deeper knowledge of adult BLS. For validity, this questionnaire was given a content validity review by faculty members of the Nursing and Midwifery School, emergency medicine specialists, and nurses with experience in cardiopulmonary resuscitation; their suggestions were then applied. The reliability of this questionnaire was confirmed with a Kuder-Richardson test result of 0.75.

Adult CPR Skills Assessment: This assessment involved two components: a questionnaire and a practical skills demonstration on a CPR mannequin.

Adult CPR Skills Assessment Questionnaire: This research-made questionnaire includes 17 questions about scene safety, responsiveness, contacting emergency services, exposing the chest, hand positioning, applying appropriate depth of chest compressions, achieving the proper number and speed of compressions, allowing the chest to recoil during massage, opening the airway with proper technique, delivering two successive artificial breaths, assessing the correctness of ventilation, the 30:2 compression-to-ventilation ratio, using AED, post-resuscitation recovery position, checking the breath for at least 5 and not more than 10 s, pulse checks within the same timeframe, and skill assessment in performing basic cardiopulmonary resuscitation operations. For certain items of high importance, such as having the correct angle of hands, performing chest massage at appropriate depth and rate, and establishing the airway correctly, a coefficient of two is considered.

For the practical skills demonstration, each participant was required to perform CPR on a mannequin equipped with a CPR meter. This mannequin automatically recorded essential parameters such as correct compression rate, number of correct chest compressions, number of correct ventilations, ventilation rate, average tidal volume, average compression depth, average compression rate, correct body positioning, and hand-off time. These parameters were synchronized with a computer and assessed using a checklist.

The scoring method is designed so that each item in the checklist is awarded zero or one point, with zero indicating lack of skill and one indicating proficiency in the relevant area. Thus, participants’ skills levels are determined and categorized. The total score for this checklist is 21. The validity of this questionnaire was also obtained through content validity, where faculty members of the Nursing and Midwifery School, emergency medicine specialists, and experienced nurses in cardiopulmonary resuscitation reviewed and provided feedback on the questionnaire. Its reliability was calculated and confirmed with a Kuder-Richardson test yielding 0.78.

### Sample size

The sample size for this research was calculated using the formula for comparing means with a 95% confidence level and an 80% test power, resulting in 30 individuals for each group.

### Data collection

Initially, upon obtaining ethical approval, the researchers visited the administration office of the Nursing and Midwifery School at Torbat Heydariyeh University of Medical Sciences and compiled a list of nursing students above the fifth semester. The nursing students entered the study in a convenient sampling manner after obtaining approval from the administration office and written consent and based on the inclusion criteria.

Before the intervention, nursing students were required to fill out a questionnaire including demographic questions and complete a pre-test to assess their knowledge and awareness of Basic Life Support (BLS) principles. Following that, a four-hour theoretical session led by the first author, based on the 2022 guidelines of the American Heart Association (AHA), was conducted for all nursing students. At the end of this phase, the nursing students were randomly assigned to two teaching groups — video feedback and infographics — using software-generated randomization in SPSS.

In the infographic group, the nursing students first attended an instructional session on the concept and uses of infographics. Subsequently, all nursing students joined a Telegram group created for this purpose. The first author of the paper was the administrator of this Telegram group.

In this group, over the course of four nights, at 8 PM each evening, educational contents on cardiopulmonary resuscitation skills in the form of infographics were sent to the nursing students, and they had an hour to discuss and ask questions in the group. The decision to conduct these sessions at 8 PM was based on the nursing students’ suggestions.

In the video feedback group, an initial two-hour session of practical CPR skills training on mannequins was conducted, followed by each nursing student was given three minutes to practice CPR on a mannequin while being recorded. After the practice session, the investigator/CPR expert reviewed the recorded video and provided subjective feedback to the nursing student, highlighting areas for improvement and offering guidance on proper technique. The nursing student then had another opportunity to practice CPR on the mannequin, incorporating the feedback received.

Both the infographic and video feedback groups were provided with sufficient time to practice CPR techniques on the mannequin during the training sessions. The infographic group had dedicated practice sessions on the mannequin, ensuring equal opportunities for hands-on practice as the video feedback group.

One week after the intervention in both the infographic and video feedback groups, nursing students’ knowledge and awareness were evaluated using a questionnaire focused on the assessment of Adult BLS knowledge. Additionally, their CPR skills were examined through a separate questionnaire dedicated to adult CPR skills.

### Statistical analysis

After coding and importing the data into statistical software, descriptive statistics (frequency distribution table, mean, and standard deviation) were used to describe the characteristics of the research sample. For quantitative variables, the mean and standard deviation were calculated. For qualitative variables, frequency and frequency percentages were determined. To examine the relationships between the qualitative variables in the two groups, chi-square tests were employed. To compare quantitative variables between the two groups, the normality of data distribution was assessed using the Kolmogorov-Smirnov test. Independent t-tests were employed if the variables followed a normal distribution. All statistical tests were performed using SPSS25 with a confidence level of 95% and a significance level of 0.05.

## Result

The demographic variables of the Video Feedback group (*n* = 38) compared to the Infographic group (*n* = 38) revealed no significant differences in age (mean ± SD) (21.8 ± 0.7 vs. 22.1 ± 1.0, *P* = 0.209), GPA (mean ± SD) (16.7 ± 0.8 vs. 16.9 ± 0.8, *P* = 0.313), sex distribution with 57.9% male and 42.1% female in the Video Feedback group compared to 47.4% male and 52.6% female in the Infographic group (*P* = 0.358), participation in CPR workshop (60.5% vs. 44.7% Yes, *P* = 0.642; 39.5% vs. 55.3% No), work experience (23.7% vs. 26.3% Yes, *P* = 0.791; 76.3% vs. 73.7% No), and CPR experience (31.6% vs. 36.8% Yes, *P* = 0.629; 68.4% vs. 63.2% No) (Table 1).

**Table 1:** Demographic variables of the video feedback and Infographic

Variable	Group		*P* value
	Video Feedback (38)	Infographic (38)	
Age (mean ± SD)	21.8 ± 0.7	22.1 ± 1.0	*P* = 0.209
GPA (mean ± SD)	16.7 ± 0.8	16.9 ± 0.8	*P* = 0.313
Sex *n* (%)			
Male	22 (57.9)	18 (47.4)	*P* = 0.358
Female	16 (42.1)	20 (52.6)	
participated in CPR workshop *n* (%)			
Yes	23 (60.5)	17 (44.7)	*P* = 0.642
No	15 (39.5)	21 (55.3)	
work experience *n* (%)			
Yes	9 (23.7)	10 (26.3)	*P* = 0.791
No	29 (76.3)	28 (73.7)	
CPR experience *n* (%)			
Yes	12 (31.6)	14 (36.8)	*P* = 0.629
No	26 (68.4)	24 (63.2)	

The comparison of CPR knowledge and skill scores between the Video Feedback group (*n* = 38) and the Infographic group (*n* = 38) revealed no significant difference in pre-training CPR knowledge score (14.3 ± 1.6 vs. 13.9 ± 1.5, *P* = 0.199) and pre-training CPR skill score (11.6 ± 3.5 vs. 11.9 ± 3.7, *P* = 0.659). However, Post-training, the infographic group showed a statistically significantly higher CPR knowledge score (17.0 ± 1.0) compared to the video feedback group (16.5 ± 0.9, *p* = 0.032). However, the actual numerical difference of 0.5-1 point in the knowledge scores may not be clinically meaningful or practically relevant, and the post-training CPR skill score was also significantly higher in the Video Feedback group (15.5 ± 2.4) compared to the Infographic group (13.5 ± 3.2, *P* = 0.004) (Table 2).

**Table 2:** Comparison of two groups of Video Feedback and Infographic

Variable		Group		*P*
		Video Feedback (38)	Infographic (38)	
CPR knowledge score (Mean ± SD)	Pre-training	14.3 ± 1.6	13.9 ± 1.5	0.199
	Post-training	16.5 ± 0.9	17.0 ± 1.0	0.032
CPR skill score (Mean ± SD)	Pre-training	11.6 ± 3.5	11.9 ± 3.7	0.659
	Post-training	15.5 ± 2.4	13.5 ± 3.2	0.004
Success in CPR (%)	Pre-training	14%	14%	
	Post-training	34%	22%	
Success in chest compression (%)	Pre-training	33%	33%	
	Post-training	59%	47%	
Success in deep chest compression (%)	Pre-training	36%	32%	
	Post-training	56%	39%	

Before the intervention, there were no significant differences between the Video Feedback group (*n* = 38) and the Infographic group (*n* = 38) in terms of appropriate depth of chest compression (42.1% vs. 34.2%, *P* = 0.479), appropriate number and speed of chest compressions (36.8% vs. 34.2%, *P* = 0.811), appropriate technique to open the airway (26.3% vs. 23.7%, *P* = 0.791), and the appropriateness of the angle and location of the hands (52.6% vs. 50.0%, *P* = 0.818).

After the intervention, significant differences were observed. The Video Feedback group showed a higher percentage of participants demonstrating appropriate depth of chest compression (81.6%) compared to the Infographic group (57.9%) with a *P* value of 0.045. Similarly, a higher percentage of participants in the Video Feedback group demonstrated appropriate number and speed of chest compressions (84.2%) compared to the Infographic group (55.3%) with a *P* value of 0.006. The Video Feedback group also showed a significantly higher percentage of participants demonstrating the appropriate technique to open the airway (84.2%) compared to the Infographic group (36.8%) with a *P* value of less than 0.001. Furthermore, a higher percentage of participants in the Video Feedback group demonstrated the appropriateness of the angle and location of the hands (73.7%) compared to the Infographic group (47.4%) with a *P* value of 0.019 (Table 3).

**Table 3:** Comparison of participant performance in CPR techniques between Video Feedback and Infographic groups

Variable	Group				*P* value
	Video Feedback (38)		Infographic (38)		
	Pretest	Posttest	Pretest	Posttest	
Appropriate depth of chest compression *n* (%)					
Yes	16 (42.1)	31 (81.6)	13 (34.2)	22 (57.9)	Before the intervention *P* = 0.479 After the intervention *P* = 0.045
No	22 (57.9)	7 (18.4)	25 (65.8)	16 (42.1)	
Appropriate number and speed of chest compressions *n* (%)					
Yes	14 (36.8)	32 (84.2)	13 (34.2)	21 (55.3)	Before the intervention *P* = 0.811 After the intervention *P* = 0.006
No	24 (63.2)	6 (15.8)	25 (65.8)	17 (44.7)	
Appropriate technique to open the airway *n* (%)					
Yes	10 (26.3)	32 (84.2)	9 (23.7)	14 (36.8)	Before the intervention *P* = 0.791 After the intervention *P* < 0.001
No	28 (73.7)	6 (15.8)	29 (76.3)	24 (63.2)	
The appropriateness of the angle and location of the hands *n* (%)					
Yes	20 (52.6)	28 (73.7)	19 (50.0)	18 (47.4)	Before the intervention *P* = 0.818 After the intervention *P* = 0.019
No	18 (47.4)	10 (26.3)	19 (50.0)	20 (52.6)	

## Discussion

The superior concepts of infographics and their impact on learning and memory form a significant foundation in modern education. Infographics, by effectively integrating text, images, and data, facilitate the information transfer process [17]. Mayer emphasized in his theory that learning occurs best when content is presented both auditorily and visually at the same time. Infographics adeptly implement these two indicators together [21].

The findings of Guo et al. also pertain to the notion that infographics, by providing a strong visualization process, make the understanding of content easier and deeper [22]. Another important aspect of using infographics is the ease of digestion and simplification of complex materials. Basco and Coyne present evidence that utilizing infographics can convey theoretical knowledge in aesthetically appealing and engaging formats, thus facilitating the learning process [23, 24].

This discussion aligns well with a study by Dorneles et al. that identified infographics as suitable tools for enhancing nursing student comprehension in various scientific fields. Powerful visual tools can assist nursing students through theoretical complexities for a fuller understanding of the material [20]. Similarly, the findings of Sisson et al. have revealed other dimensions of the significance of infographics in education. Their emphasis on the effectiveness of infographics in elevating nursing students’ knowledge levels shows that these tools are not only aesthetically pleasing but effective in fostering cognitive skills [25].

On the other hand, video feedback plays a significant role in the instruction of clinical and practical skills (such as cardiopulmonary resuscitation). Qadiri and colleagues suggest in their studies that immediate visual feedback significantly affects the quality of cardiopulmonary resuscitation performance. The core idea is that receiving immediate and accurate feedback enables learners to recognize their errors promptly, making the learning process faster and more precise. Consistent with these findings, the group that utilized video feedback demonstrated improved motor skills. This type of feedback allows learners to watch their performance in real-time and compare their process with an ideal model [26].

Saidu et al. have also addressed research that video-based learning not only aids in enhancing CPR skills but also in their retention over longer periods. Video feedback, due to its visual nature, has a considerable impact on creating deep learning and a better understanding of CPR processes among the learners [27]. Spence and colleagues have noted the value of video feedback and its impact on overall performance and skills related to cardiopulmonary resuscitation. Feedback presented visually is not only a means of assessment but also an opportunity for continuous learning and practice, which can lead to ongoing improvement [28]. The combination of these results indicates that the use of video feedback in CPR training can significantly enhance both practical and theoretical skills. Furthermore, improving practical skills through video feedback can help better execute CPR in real hospital environments and increase the likelihood of success in emergencies. Video feedback not only serves as a tool for assessing and enhancing the desired skills but potentially plays a vital role in transferring learned skills to clinical environments and improving the efficacy of interventions in critical situations. This has been confirmed by several different studies, and its place in modern educational programs has been increasingly emphasized [29].

It is important to note that the video feedback intervention in our study relied on subjective feedback from an investigator/CPR expert, rather than real-time CPR quality feedback based on objective metrics such as compression rate, depth, and recoil. While subjective feedback can provide valuable insights, incorporating real-time metric-based feedback may offer a more precise and objective approach to improving CPR performance.

While our study found a statistically significant difference in post-training CPR knowledge scores favoring the infographic group, the actual numerical difference was relatively small (0.5-1 point). This raises questions about the clinical significance and practical relevance of this difference in knowledge retention between the two educational approaches. Future studies should consider incorporating more comprehensive knowledge assessments and explore the long-term retention of knowledge to better evaluate the practical implications of these findings.

The study’s limitations include a sample size drawn only from a single educational institution, which may not represent all demographic backgrounds. The absence of a control group using standard CPR training limited the ability to compare new methods with traditional teaching practices. Also, A limitation of the present study is the short duration between the intervention and the assessment, which may not accurately reflect long-term skill and knowledge retention. The study’s focus on nursing students may not generalize to other health professional trainees, and self-reported measures without external validation could introduce bias.

The study has several strengths, including a randomized design that enhances the internal validity of the findings. It employed validated questionnaires to assess both knowledge and practical skills accurately. The use of video feedback and infographics responded to identified gaps in educational methods, applying modern pedagogical approaches to complex skill training. The integration of technology, specifically Telegram for infographics and video for feedback, reflects an innovative approach to learning that is in line with current digital trends.

Future research should consider larger and more diverse populations to increase generalizability. Longitudinal studies to assess long-term retention of BLS skills post-intervention will be invaluable in educational settings. Comparisons with control groups undergoing traditional training methods could contextualize the value of these interventions. Further, crossover studies where each participant experiences both interventions could control for inter-individual variability. Expanding the research to include other healthcare professionals and incorporating multidimensional learning evaluations could offer a broader understanding of educational outcomes. Integrating a hybrid model that combines the strengths of both infographic and video feedback might be an advanced step in CPR education enhancement.

## Conclusion

The research presented in this comparative study elucidates that both infographic and video feedback methodologies have beneficial effects on the enhancement of nursing students’ clinical skills in Basic Life Support (BLS). The use of infographics significantly increased the nursing students’ knowledge levels, suggesting its efficacy in theoretical retention. Conversely, video feedback substantially improved the nursing students’ practical CPR skills, indicating its vital role in the refinement of motor skills necessary for effective CPR performance.

This dual approach to learning - leveraging the cognitive engagement of infographics with the practical enhancement provided by video feedback - is indicative of a robust educational model. Consistently integrating these methods, to cater to both theoretical understanding and practical application, could substantially elevate the quality of BLS instruction. This integration may consequently increase the effectiveness of life-saving interventions performed by future healthcare professionals.

## Data Availability

The datasets generated in the current study are available from the corresponding author upon reasonable request.
